# Social buffering of human fear is shaped by gender, social concern, and the presence of real vs virtual agents

**DOI:** 10.1038/s41398-021-01761-5

**Published:** 2021-12-20

**Authors:** Yanyan Qi, Dorothée Bruch, Philipp Krop, Martin J. Herrmann, Marc E. Latoschik, Jürgen Deckert, Grit Hein

**Affiliations:** 1 Center of Mental Health, Department of Psychiatry, Psychosomatic and Psychotherapy, Translational Social Neuroscience Unit, University of Wurzburg, 97080 Wurzburg, Germany; 2 Department of Psychology, School of Education, Zhengzhou University, 450001 Zhengzhou, China; 3 Human-Computer Interaction, University of Wurzburg, Am Hubland, 97074 Wurzburg, Germany

**Keywords:** Human behaviour, Physiology

## Abstract

The presence of a partner can attenuate physiological fear responses, a phenomenon known as social buffering. However, not all individuals are equally sociable. Here we investigated whether social buffering of fear is shaped by sensitivity to social anxiety (social concern) and whether these effects are different in females and males. We collected skin conductance responses (SCRs) and affect ratings of female and male participants when they experienced aversive and neutral sounds alone (alone treatment) or in the presence of an unknown person of the same gender (social treatment). Individual differences in social concern were assessed based on a well-established questionnaire. Our results showed that social concern had a stronger effect on social buffering in females than in males. The lower females scored on social concern, the stronger the SCRs reduction in the social compared to the alone treatment. The effect of social concern on social buffering of fear in females disappeared if participants were paired with a virtual agent instead of a real person. Together, these results showed that social buffering of human fear is shaped by gender and social concern. In females, the presence of virtual agents can buffer fear, irrespective of individual differences in social concern. These findings specify factors that shape the social modulation of human fear, and thus might be relevant for the treatment of anxiety disorders.

## Introduction

Excessive fear responses are characteristic of anxiety disorders and pave the way for a variety of other psychological and psychiatric problems. There is evidence from animal models that autonomic [1, 2] and neural [2–5] fear responses are reduced in the presence of a non-fearful conspecific, a phenomenon known as social buffering [6–8]. Adapting the paradigms that were used to study social buffering in animals, recent studies have shown similar social buffering of autonomic fear or stress responses in humans [9–11], in particular in individuals who score higher on trait [12, 13] or state [9, 14] anxiety.

Social buffering effects in animals and humans are assumed to occur, because the presence of a conspecific or another individual signals the availability of resources to cope with imposed threat, and thus is perceived as a safety cue [11, 15, 16]. On the neurobiological level, the presence of a conspecific has been shown to reduce stress-induced changes in neural circuitries [2, 11, 17, 18] and synapses [3, 4].

However, not all humans are equally sociable. In some individuals, the presence of another person induces concern and anxiety, paralleled by an increase in autonomic fear and hormonal stress responses [19–21]. The sensitivity to anxiety in social situations has been defined as an independent dimension of human anxiety, known as social concern [22–25]. Social concern is characterized by worries about publicly observable anxious reactions such as trembling, blushing, or sweating in front of others [22, 26]. As a result, for individuals scoring high on social concern, the presence of another individual may impose a threat, instead of a safety cue that may trigger social buffering.

There is recent evidence suggesting that the virtual presence of another person can reduce responses to social stress, if participants believe that the virtual agent was steered by a real person [27, 28]. Based on these findings, it is possible that similar to a real person the presence of a virtual person can be perceived as a safety cue and thus elicits social buffering effects, and is irrespective of individual social concern.

Social anxiety is known to manifest differently in females and in males. In females, social anxiety tends to be generalized, i.e., to occur in a variety of different social contexts [29, 30]. In contrast, males are mainly concerned and anxious in situations related to mating, indicated by increased physiological fear responses in the presence of a potential partner [31]. Given that other individuals are seen as a threat in many different social contexts in females and only in mating contexts in males, high sensitivity to social anxiety may have a stronger effect on social buffering in females than in males.

Taken together, there is evidence that the effects of social buffering can attenuate physiological responses in humans, and thus are potentially relevant for the diagnosis and treatment of anxiety disorders. However, it remains unclear as to whether and how social buffering of human fear is shaped by social concern, whether the effects of social concern are different in females and males, and whether and how social buffering effects differ in the presence of a real and a virtual person.

**Fig. 1:**
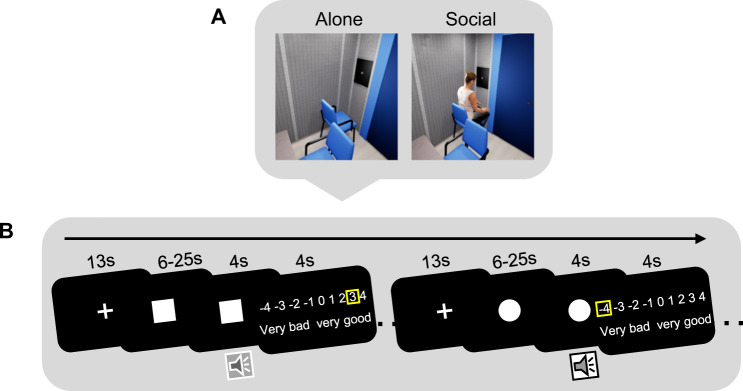
Visualization of the experimental set up in the virtual reality (VR) study including the virtual agent and example trial sequence. **A** The virtual environment in the VR study was an exact replication of the testing room used in the real-life study. The virtual agent was created based on a body scan of the person that was present in the real-life study. **B** Participants were presented with abstract symbols that were associated with an aversive or a neutral sound. After the presentation of the sound, they rated how they felt on a scale from −4 (very bad) to +4 (very good).

To address these questions, we first tested how social concern influences the social buffering of fear in the presence of an unknown person in females and males (real-life; Study 1). Second, we investigated the effect of social concern on social buffering in the presence of a virtual agent (virtual reality, VR; Study 2). The agent was an exact virtual representation (created based on a body scan) of the real partner in Study 1 and was placed in a virtual environment that was identical to the testing room used in the real-life study (Fig. 1A). It was clear to the participants that the agent was not steered by a real person.

In more detail, in the real-life and the VR studies, participants were presented with the same aversive, fear-inducing or neutral sounds, and their autonomic responses (skin conductance responses, SCRs) and subjective ratings were measured. Participants experienced the sounds alone (real-life/VR alone treatment groups), or in the presence of an unknown person of the same gender and ethnicity, or a virtual representation of that person (real-life/VR social treatment groups). The real or virtual partners faced away from the participant and her or his screen and fixated on the opposite wall (Fig. 1A). This manipulation was important to minimize social interaction and the effect of social evaluation. Hence, the partner was merely physically present, without direct social interaction, similar to studies that investigated social buffering in animals. The gender of the partner (confederate) matched the gender of the participants to avoid the potential complications inherent in mixed-gender pairings and consistent with same-sex setting used by most animal studies [32, 33].

We assessed individual differences in social concern (worries regarding social censure or rejection because of publicly observable reactions) [22, 23] using the Anxiety Sensitivity Index (ASI-3; 22). The ASI-3 is a well-established questionnaire that allows for assessing social concern in parallel with other anxiety dimensions, i.e., physical and cognitive concern (see below for details). The parallel assessment of different anxiety dimensions is important, because it allows us to evaluate the effect of social concern, compared to physical and cognitive concern.

In more detail, the social concern scale assesses concern regarding observable reactions in the presence of others. Individuals indicate whether they are concerned to appear nervous (“It is important for me not to appear nervous.”), tremble (“When I tremble in the presence of others, I fear what people might think of me.”), blush (“It scares me when I blush in front of people.”), sweat (“When I begin to sweat in a social situation, I fear people will think negatively of me.”) or faint (“I think it would be horrible for me to faint in public.”) in the presence of another person. Accordingly, social concern can be elicited by the mere presence of another individual.

Guided by previous studies [9, 34], we assumed that the real or virtual presence of another person might reduce the subjective and autonomic fear responses, indicating a social buffering effect. In this case, the SCRs and subjective ratings to aversive sounds should be significantly smaller in the social compared to the alone treatment groups. Given that individuals scoring high on social concern tend to fear observable reactions in the presence of others (e.g., blushing, trembling etc), high social concern scores should counteract social buffering. The higher the social concern, the smaller the reduction in SCRs and subjective ratings to aversive sounds in the social compared to the alone treatment groups. Inspired by findings showing more generalized social anxiety effects in females [29–31], we hypothesized that this effect might be stronger in females than in males, reflected by a significant interaction between gender (female/male), treatment (alone/social) and social concern. Motivated by studies showing a reduction of social stress during virtual compared to real-life social interactions [35], we expected that the effect of social concern on social buffering should be significantly stronger in the presence of a real person compared to the presence of a virtual representation (agent) of this same person.

## Methods and materials

### Real-life study 1

#### Participants

The sample size required to detect differential effects between treatment groups and the modulating effect of individual differences (here social concern) was estimated based on a power calculation (threshold of 80% sensitivity (1 − beta = 0.80); *α* = 0.05; medium effect size [36]) of our previous study [9] in which we used a similar design as in the current study. The results yielded a required sample size smaller than 26 in each group. In total, 147 healthy Caucasian participants (72 females) participated in this study. The participants had no history of neurological or psychiatric disorders and were pre-screened to exclude depressive symptoms [37]. All participants gave written informed consent before the study. Participants were randomly assigned to an alone treatment group or a social treatment group. The participants in the social treatment group were paired with a person of the same gender and ethnicity, i.e., a Caucasian female or male student who was unknown to all the participants, to avoid the potential complications of gender-mixed and ethnicity-mixed pairings of participants and confederates. The confederate was asked to face away from the participant and her or his screen and to fixate on the opposite wall to minimize social interaction and the effect of social evaluation. Participants in the alone treatment group completed the experiment in absence of another person (Fig. 1A). The data of 13 participants had to be excluded: four due to technical problems, six because of extensive artefacts in the SCRs signal that could not be corrected, and three because of a lack of SCRs during the entire data acquisition. We analyzed the data of 134 participants: 31 females in the alone treatment (*M*_age_ = 23.74, SD_age_ = 0.40), 35 females in social treatment (*M*_age_ = 25.51, SD_age_ = 0.85), 34 males in alone treatment (*M*_age_ = 24.76, SD_age_ = 0.72) and 34 males in social treatment (*M*_age_ = 27.21, SD_age_ = 0.86). The data of the female sample were part of an analysis reported elsewhere [9]. However, none of the analyses in the current manuscript have been previously reported. The study was approved by the ethics committee of the University Hospital of Wurzburg (142/18), and all procedures were conducted in accordance with the Helsinki Declaration of 1975 and its later amendments.

#### Scales

Before the study, all participants were screened for depression [37] and filled in questionnaires measuring different dimensions of anxiety sensitivity [22], perceived social support (Multidimensional Scale of Perceived Social Support, MSPSS; [38]), positive and negative affect (Positive and Negative Affect Schedule, PANAS; [39]) and state and trait anxiety (State-Trait Anxiety Inventory, STAI; [40]). After the study, participants of the social treatment groups rated statements regarding their impressions of the other person (the confederate) on a well-established scale [41], ranging from 1 (not at all) to 9 (very much).

#### Stimuli and paradigm

During the test, all participants were presented with aversive and neutral sounds via headphones. The sounds were taken from the International Affective Digital Sounds system (IADS; [42]). Previous studies showed that the presentation of the aversive sounds can induce fear, which was significantly stronger than the neutral sounds [9, 43]. The type of the sound was indicated by a preceding image (cue) presented on a black screen: a white circle was the cue for aversive sounds whereas a white square indicated neutral sounds. The sounds and corresponding images were presented in a pseudo-randomized order. Each trial started with a fixation cross, which was replaced by one of the cues. After an interval varying from 6 to 25 s (mean interval = 16.2 s), the respective sound (4 s) was presented. Then participants rated their feeling while hearing the sound on a continuous scale from −4 (very bad) to +4 (very good) that was presented for 4 s (Fig. 1B). The cursor of the rating scale was placed at a random position in each trial to keep participants’ attention and minimize rating error. There were three blocks of 18 trials with half of them including aversive sounds and the remaining half of them including neutral sounds. Before the main experiment, all participants performed four practice trials: two containing aversive sounds and two containing neutral sounds to familiarize themselves to the task.

#### Recording of SCRs

The SCRs were continuously collected during the main part of the experiment by a Brainamp ExG MR amplifier (Brain Products GmbH), and recorded by Vision Recorder software (Brain Products GmbH). Two Ag/AgCl electrodes filled with non-hydrating gel were attached to the thenar and hypothenar of participants’ nondominant palm. The sample rate was 1000 Hz. The constant voltage across the two electrodes was 0.5 V [9].

#### Data analysis

The SCRs were pre-processed using the EEGLAB toolbox [44]. Low-pass filtering (1 Hz) was performed on the raw data to remove high-frequency noise. The cue and the sound onset were defined as 0 s. For each trial, an epoch during the cue presentation (time window −1 to 6 s), and an epoch during sound presentation period (time window −1 to 10 s) were defined and separately segmented. The responses during the 1 s before the stimulus were used for baseline correction. Based on the grand-averaged wave, the SCRs value was characterized as the area under the curve per second in the time window from 2.5 to 6 s after stimulus onset, a method that has been recommended if discrete SCRs are hard to discern in one experimental condition (here discrete SCRs in response to the neutral cues/sounds were hard to detect) [9, 45–47].

Given that the cue-related SCRs did not differ between genders and studies (Tables S1, S2) and did not influence the observed social buffering effects (Tables S3–S6), our main analyses focused on SCRs elicited by the sound presentation. SCRs were transformed by log (x + 1) to correct for skewness.

We conducted linear mixed models with trial by trial affect ratings and SCRs as the dependent variable to investigate the buffering effect (LMM, “lme4” package of the R-software; [48]). We tested all fixed effects by setting a different “baseline” value for each participant (random intercept) to resolve non-independence of SCRs/affect ratings for each participant. The variables were included into the model based on our primary hypotheses. When testing the higher order interaction, all lower order terms were included in the regression equation [49]. All continuous predictors had been normalized to avoid potential multicollinearity [49]. To control for unspecific effects of state anxiety, depression and negative emotions before the experiment, the individual scores of the depression scale [23], PANAS-negative emotion scale [24], and the STAI-state-pre anxiety [10] were included as covariates in all LMMs. There was a significant overall decline in SCRs to aversive sounds in the second compared to the first half of the experiment. However, this habituation effect did not affect the observed social modulation effects (Tables S7, S8).

Effect sizes reported for LMM results are based on the marginal *R*^*2*^ *(R*^*2*^_*m*_*)* which estimates the proportion of variance explained by the fixed factors [50], calculated with the R package “MuMIn” [51]. Effect sizes reported for post-hoc and simple effect tests are based on estimated marginal means (EMM) [52], calculated with the R package “emmeans”. The Kenward–roger method was used for freedom degree estimation.

### VR study 2

#### Participants

Sixty-one healthy Caucasian females participated in the VR study. The inclusion criteria were the same as in Study 1. Participants were randomly assigned to the alone and social treatment groups. The data of ten participants had to be excluded: one due to technical problems, one because of extensive artefacts in the SCRs signal that could not be corrected, six because of lack of SCRs, and two because of problems conducting the ratings. In total, we analyzed the data of 51 participants: 25 females in the alone treatment group (*M*_age_ = 23.29, SD_age_ = 0.42) and 26 females in social treatment group (*M*_age_ = 23.98, SD_age_ = 0.57). As Study 1, Study 2 was approved by the ethics committee of the University Hospital of Wurzburg. All participants gave written informed consent before the study. All procedures were conducted in accordance with the Helsinki Declaration of 1975 and its later amendments.

#### Scales

Participants completed the same scales as in Study 1. In addition, we used a German version of the Networked Minds Measure of Social Presence (NMMSP; [53]) to test how participants perceived the presence of the agent. It has two sub-dimensions, measuring perceived co-presence and psycho-behavioral accessibility. The co-presence scale measures the degree to which the presence of the agent is processed on a low-level sensory level (e.g., I was often aware of my partner in the room). The psycho-behavioral interaction scale measures the degree of perceived social interactions with the virtual agent (e.g., I was able to communicate my intentions clearly to my partner) on a scale from ‘1’ (not at all) to ‘7’ (very much).

#### Stimuli and paradigm

The stimuli and the paradigm were same as in Study 1 (Fig. 1B), except that participants performed the study in a virtual environment. They wore a VR headset (HTC Vive Pro). The resolution of the goggles was 2880 × 1600 pixels. The virtual environment was a precise representation of the testing room used during data collection in Study 1 (Fig. 1A), which was created with Unreal Engine 4.24.3. The agent was created based on a high-resolution body scan of the female confederate that was present in Study 1, following a human avatar generation pipeline by Achenbach et al. [54]. The confederate had given consent to publish the avatar picture for scientific use. As the confederate, the virtual agent faced away from the participant (Fig. 1A) and performed small humanoid movements (e.g., slightly moving the arm on the arm rest of the chair), but did not interact with the participant. The affect ratings were conducted by pressing the button on the controller.

#### Recording of SCRs and data analyses

Recording and pre-processing of the SCRs was identical to Study 1.

To investigate differences in social buffering effects between real and virtual social presence and the modulating effect of social concern, we combined the female data in the real-life (female data in Study 1) and the data in the VR (Study 2). Study (real-life/VR) was included as a between-subject variable. The statistics of SCRs were identical to Study 1.

## Results

### Study 1: Testing the effect of social concern on social buffering in the presence of a real person

#### Scales

The Cronbach’s α coefficients of scales ranged from 0.60 to 0.92 (see Table 1 for details). The average scores on ASI-3, MSPSS, ADS, STAI, and PANAS were comparable between the four groups (female-alone; female-social; male-alone; male-social) (Table 1). The only difference was found in impression ratings for the present partner that were more positive in the female compared to the male sample (Table 1).

**Table 1:** Characteristics of the participants in Study 1.

	Female-alone mean (SE)	Female-social mean (SE)	Male-alone mean (SE)	Male-social mean (SE)	*F*/*t*-test (p)	Cronbach’s α
ASI-3						
- Social concern	8.90 (0.76)	8.97 (0.68)	8.94 (0.68)	8.94 (0.71)	0.002 (0.96)	0.73
- Physical concern	5.61 (0.79)	5.77 (0.68)	5.68 (0.70)	4.68 (0.67)	0.67 (0.42)	0.78
- Cognitive concern	4.23 (0.61)	4.34 (0.68)	4.09 (0.48)	4.26 (0.65)	0.002 (0.96)	0.77
MSPSS						
- Family	24.03 (0.98)	23.71 (0.96)	23.91 (0.76)	23.61 (0.67)	0001 (0.99)	0.92
- Friends	25.13 (0.76)	25.40 (0.51)	24.68 (0.53)	24.44 (0.63)	0.17 (0.68)	0.89
- Significant other	25.64 (0.78)	25.94 (0.59)	24.79 (0.50)	24.35 (0.64)	0.34 (0.56)	0.89
ADS	7.52 (0.83)	7.66 (0.74)	5.91 (0.62)	6.56 (0.70)	0.12 (0.73)	0.73
STAI-trait	35.10 (1.29)	35.29 (1.53)	32.82 (0.90)	34.97 (1.56)	0.52 (0.47)	0.89
STAI-state-pre	33.57 (1.27)	34.03 (1.27)	33.65 (0.78)	34.00 (1.21)	0.002 (0.96)	0.85
STAI-state-post	40.06 (1.52)	40.66 (1.57)	38.62 (1.47)	40.47 (1.70)	0.16 (0.69)	0.91
PANAS-positive	29.47 (0.98)	28.77 (1.16)	3.97 (0.87)	31.26 (1.10)	0.23 (0.64)	0.83
PANAS-negative	11.40 (0.40)	11.54 (0.25)	11.62 (0.30)	12.21 (0.39)	0.44 (0.61)	0.60
Impression ratings						0.79
- Pleasant		6.09 (0.23)		5.21 (0.22)	**2.80 (0.007)**	
- Sympathetic		6.51 (0.25)		5.71 (0.24)	**2.36 (0.02)**	
- Helpful		4.06 (0.39)		2.56 (0.40)	**2.71 (0.009)**	
- Make the task easier		3.92 (0.41)		2.89 (0.37)	**1.84 (0.070)**	

Bold values are significant at *p* < 0.05.

*ASI-3* Anxiety Sensitivity Index-3, *MSPSS* Multidimensional Scale of Perceived Social Support, *ADS* Allgemeine Depressions Skala, *STAI* State-Trait Anxiety Inventory, *PANAS* Positive and Negative Affect Schedule, *SE* standard error.

#### Aversive sounds elicited a more negative affect and larger SCRs than neutral sounds

As a manipulation check, we compared the affect ratings and SCRs to aversive and neutral sounds in the alone treatment groups. The results showed significantly more negative affect ratings for aversive compared to neutral sounds in females and males, with a stronger effect in females, *B* = −0.56, *p* < 0.001 (females: aversive sounds, *M* = −1.97, SE = 0.11, neutral sounds, *M* = 1.33, SE = 0.11, *t*(3443) = −56.28, *p* < 0.0001, EMM = −3.31, SE = 0.059; males: aversive sounds, *M* = −1.69, SE = 0.11, neutral sounds, *M* = 1.05, SE = 0.11, *t*(3443) = −48.91, *p* < 0.0001, EMM = −2.74, SE = 0.056).

Moreover, SCRs to aversive sounds were significantly higher than neutral sounds with a stronger effect in females, *B* = 0.083, *p* < 0.001 (females: aversive sounds, *M* = 0.15, SE = 0.010, neutral sounds, *M* = −0.0063, SE = 0.010, *t*(3420) = 18.41, *p* < 0.001, EMM = 0.16, SE = 0.0084; males: aversive sounds, *M* = 0.084, SE = 0.010, neutral sounds, *M* = 0.012, SE = 0.010, *t*(3420) = 8.97, *p* < 0.001, EMM = 0.072, SE = 0.0080).

The SCRs to aversive sounds were significantly related to participants’ affect ratings with a larger effect in females, *B* = 0.024, *p* < 0.001 (females: *B* = −0.057, 95% CI = [−0.063, −0.052], males: *B* = −0.033, 95% CI = [−0.039, −0.027]). The higher the SCRs to the aversive sounds, the more negative participants’ affect ratings.

#### Social buffering in females, but not in males

First, we tested if the presence of a real partner had an effect on the ratings that participants made after hearing the sounds. An LMM with affect ratings to aversive sounds as the dependent variable and treatment (alone/social), gender (female/male) and treatment × gender as the predictors showed no significant effects, treatment (*B* = 0.35, *p* = 0.16), gender (*B* = 0.32, *p* = 0.21), treatment × gender (*B* = −0.21, *p* = 0.56); *R*^*2*^_*m*_ = 0.028.

**Fig. 2:**
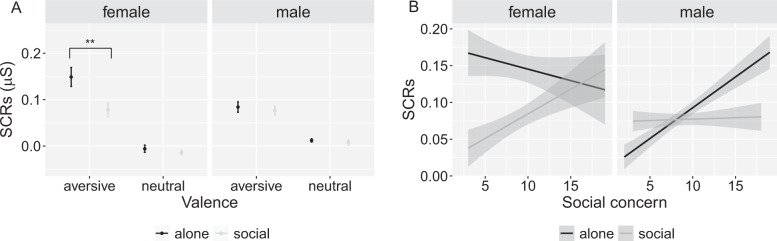
Average skin conductance responses (SCRs) and effect of social concern in female and male participants. **A** Gender effect on average SCRs to aversive and neutral sounds in the alone and the social treatment groups, showing a significant social buffering effect in females, but not in males. **B** Interaction between treatment (alone/social), gender (female/male) and individual social concern scores, reflecting a stronger effect of social concern on SCRs to aversive sounds in females compared to males. The values of SCRs were transformed by log (x + 1) to correct the skewness. Error bars indicate standard error. The shade area in (**B**) indicates 95% confidence interval. **p < 0.01.

Second, we performed the same model with SCRs to aversive sounds as the dependent variable. The results showed significant main effects of treatment (*B* = −0.064, *p* = 0.0017) and gender (*B* = −0.058, *p* = 0.006), and a significant treatment × gender interaction (*B* = 0.059, *p* = 0.042) (Fig. 2A; Table 2); *R*^*2*^_*m*_ = 0.019. Clarifying the interaction effect, simple effect analyses showed significantly larger SCRs in the alone treatment group compared to the social treatment group in females (alone: *M* = 0.14, SE = 0.015, social: *M* = 0.077, SE = 0.014, *t*(126) = 3.13, *p* = 0.0022, EMM = 0.064, SE = 0.021), but not in males (alone: *M* = 0.084, SE = 0.014, social: *M* = 0.078, SE = 0.014, *t*(126) = 0.29, *p* = 0.77, EMM = 0.0058, SE = 0.020). Together, these SCRs results indicate a significant social buffering effect in the female sample, but not in the male sample.

**Table 2:** Results of LMM testing gender effects in social buffering.

	*B*	SE	*χ*^*2*^	Df	*P value*
(Intercept)	0.14	0.015	86.05	1	**<0.001**
Treatment	−0.064	0.021	9.82	1	**0.0017**
Gender	−0.058	0.021	7.56	1	**0.006**
PANAS-negative	−0.0039	0.0093	0.17	1	0.68
STAI-state-pre	0.0005	0.0099	0.0029	1	0.96
ADS	−0.0004	0.0091	0.0021	1	0.96
Treatment × Gender	0.059	0.029	4.14	1	**0.042**

Bold values are significant at *p* < 0.05.

*PANAS* Positive and Negative Affect Schedule, *STAI-state-pre* state anxiety scores before the experiment based on the State scale of the State-Trait Anxiety Inventory (STAI), *ADS* Allgemeine Depressions Skala, *SE* standard error.

Given the gender differences in impression ratings (Table 1), it is possible that females showed stronger social buffering because they perceived their partner more positively than males. In this case, the individual differences in impression ratings should significantly predict the individual differences in SCRs in the social treatment groups. Note that this analysis can only be conducted on the social treatment groups, because participants in the alone treatment groups did not have a partner and thus could not provide impression ratings. An LMM with SCRs to aversive sounds as the dependent variable and gender, impression rating (the sum value), and gender × impression rating as the predictors showed no significant main effects of gender (*B* = 0.0035, *p* = 0.87) and impression rating (*B* = 0.0098, *p* = 0.47), and no significant gender × impression rating interaction (*B* = −0.012, *p* = 0.54); *R*^*2*^_*m*_ = 0.0089. According to this result, gender differences in impression ratings are unlikely to account for the observed gender difference in social buffering.

#### Differential effects of social concern on social buffering in females and males

To investigate the effect of social concern on social buffering in females and males, we conducted an LMM with social concern, gender (female/male), treatment (social/alone) and their interaction terms as the predictors and SCRs to aversive sounds as the dependent variable. The results showed significant main effects of gender (*B* = −0.056, *p* = 0.007) and treatment (*B* = −0.064, *p* = 0.001), significant two-way interactions between social concern and gender (*B* = 0.051, *p* = 0.011), between treatment and gender (*B* = 0.059, *p* = 0.036), and between treatment and social concern (*B* = 0.044, *p* = 0.027), and a significant three-way interaction between social concern, treatment and gender (*B* = −0.077, *p* = 0.007), *R*^*2*^_*m*_ = 0.034 (Fig. 2B and Table 3).

**Table 3:** Results of the LMM testing the effect of social concern on buffering in different genders.

	*B*	SE	*χ*^*2*^	Df	*P value*
(Intercept)	0.14	0.015	88.56	1	**<0.001**
Treatment	−0.064	0.020	1.21	1	**0.001**
Gender	−0.056	0.021	7.34	1	**0.007**
Social concern	−0.016	0.014	1.31	1	0.25
PANAS-negative	−0.0083	0.0092	0.81	1	0.37
STAI-state-pre	0.0015	0.0098	0.024	1	0.88
ADS	0.0029	0.0091	0.100	1	0.75
Treatment × Gender	0.059	0.028	4.42	1	**0.036**
Treatment × Social concern	0.044	0.020	4.89	1	**0.027**
Gender × Social concern	0.051	0.020	6.33	1	**0.012**
Treatment × Gender × Social concern	−0.077	0.028	7.36	1	**0.0067**

Bold values are significant at *p* < 0.05.

*PANAS* Positive and Negative Affect Schedule, *STAI-state-pre* state anxiety scores before the experiment based on the State scale of the State-Trait Anxiety Inventory (STAI), *ADS* Allgemeine Depressions Skala, *SE* standard error.

To unpack the three-way interaction, we conducted an LMM separately for females and males. For females, the result showed a significant main effect of treatment (*B* = −0.064, *p* = 0.010), and a marginally significant social concern × treatment effect (*B* = 0.046, *p* = 0.066); *R*^*2*^_*m*_ = 0.028. As shown in Fig. 2B, in the social treatment group, females showed a reduction in SCRs to aversive sounds with decreasing social concern (*B* = 0.036, *p* = 0.014), while there was no such an effect in the alone treatment group (*B* = −0.025, *p* = 0.22). In males we found a significant main effect of social concern (*B* = 0.034, *p* = 0.0017), and a significant social concern × treatment interaction (*B* = −0.030, *p* = 0.045); *R*^*2*^_*m*_ = 0.036. In males, the interaction effect was driven by a positive relationship between social concern and SCRs to aversive sounds in the alone treatment group (*B* = 0.030, *p* = 0.0094), while there was no such effect in the social treatment group (*B* = 0.0045, *p* = 0.68).

To test if the observed effects are specific for social concern, we conducted the same analyses with the two other anxiety dimensions of the ASI, i.e., physical concern and cognitive concern. The respective LMMs revealed no significant three-way interactions (physical concern × treatment × gender: *B* = −0.0075, *p* = 0.80, cognitive concern × treatment × gender: *B* = 0.021, *p* = 0.50; Tables S9 and S10).

### Study 2: Testing the effect of social concern on social buffering in the presence of a virtual person

Study 1 revealed that females showed a reduction in fear-related SCRs in the presence of another person, but only if they scored low on social concern. However, it is unclear if social concern counteracts social buffering in females in general, or just in the presence of a real person. If social concern reduces social buffering only in the presence of a real person, the presence of a virtual person should induce social buffering in females irrespective of social concern. Study 2 tested this assumption by investigating the effect of social concern on social buffering of fear in females in the presence of a virtual person (agent).

#### Scales

The questionnaire scores of the female participants in Studies 1 and 2 were comparable (Table 4). The Cronbach’s α coefficients of these questionnaires ranged from 0.70 to 0.93 (see Table 4 for details). The results of the NMMSP questionnaire [53] indicated that the virtual agent induced a moderate level of perceived co-presence (*M* = 3.54, SE = 0.15) and a low level of perceived social interactions (*M* = 1.39, SE = 0.013). The impression ratings for the real partner were more positive than for the virtual agent (Table 4).

**Table 4:** Characteristics of the participants in Study 2.

	Real-life-alone Mean (SE)	Real-life-social Mean (SE)	VR-alone Mean (SE)	VR-social Mean (SE)	*F*/*t*-test	Cronbach’s α
ASI-3						
- Social concern	8.90 (0.76)	8.97 (0.68)	9.88 (1.06)	9.81 (0.86)	0.007 (0.93)	0.77
- Physical concern	5.61 (0.79)	5.77 (0.68)	5.44 (0.92)	6.31 (1.03)	0.18 (0.68)	0.82
- Cognitive concern	4.23 (0.61)	4.34 (0.68)	4.52 (0.71)	5.77 (0.90)	0.61 (0.44)	0.82
MSPSS						
- Family	24.03 (0.98)	23.71 (0.96)	24.48 (0.92)	24.23 (0.71)	0.001 (0.97)	0.93
- Friends	25.13 (0.76)	25.40 (0.51)	25.04 (0.72)	26.15 (0.48)	0.44 (0.51)	0.91
- Significant other	25.64 (0.78)	25.94 (0.59)	26.20 (0.85)	26.11 (0.58)	0.072 (0.79)	0.93
ADS	7.52 (0.83)	7.66 (0.74)	8.48 (1.03)	7.85 (0.97)	0.19 (0.66)	0.77
STAI-Trait	35.10 (1.29)	35.29 (1.53)	34.44 (1.32)	35.54 (1.62)	0.095 (0.76)	0.89
STAI-state-pre	33.57 (1.27)	34.03 (1.27)	38.52 (1.69)	37.42 (1.60)	0.29 (0.59)	0.89
STAI-state-post	40.06 (1.52)	40.66 (1.57)	42.76 (2.18)	40.35 (1.81)	0.73 (0.40)	0.92
PANAS-positive	29.47 (0.98)	28.77 (1.16)	31.20 (1.50)	33.58 (1.24)	1.58 (0.21)	0.87
PANAS-negative	11.40 (0.40)	11.54 (0.25)	13.64 (0.58)	13.38 (0.59)	0.20 (0.66)	0.70
Impression ratings						0.83
- Pleasant		6.09 (0.23)		3.57 (0.23)	**6.45 (<0.001)**	
- Sympathetic		6.51 (0.25)		3.80 (0.29)	**6.75 (<0.001)**	
- Helpful		4.06 (0.39)		2.78 (0.32)	**2.54 (0.013)**	
- Make the task easier		3.92 (0.41)		2.69 (0.30)	**2.48 (0.015)**	

Bold values are significant at *p* < 0.05.

*ASI-3* Anxiety Sensitivity Index-3, *MSPSS* Multidimensional Scale of Perceived Social Support, *ADS* Allgemeine Depressions Skala, *STAI* State-Trait Anxiety Inventory, *PANAS* Positive and Negative Affect Schedule, *SE* standard error.

#### Aversive sounds elicited a more negative affect and larger SCRs than neutral sounds

As in Study 1, participants showed more negative ratings to aversive sounds compared to neutral sounds (aversive sounds, *M* = −2.23, SE = 0.15, neutral sounds, *M* = 1.93, SE = 0.15, *t* (2966) = −6.90, *p* < 0.001, EMM = −4.16, SE = 0.068). They also showed larger SCRs to aversive sounds compared to neutral sounds (aversive sounds, *M* = 0.086, SE = 0.013, neutral sounds, *M* = 0.0011, SE = 0.013, *t* (2942) = 8.43, *p* < 0.001, EMM = 0.085, SE = 0.010). Moreover, as in Study 1, there was a significant negative relationship between SCRs and affect ratings (*B* = −0.032, 95% CI = [−0.038 −0.026]), indicating that an increase in SCRs reflected a more negative emotional state.

#### Social buffering of fear in females in the presence of a real and a virtual person

We investigated if the mere presence of a virtual agent had a similar buffering effect on fear responses as the mere presence of a real person. First, we performed an LMM with affect ratings to aversive sounds as the dependent variable and treatment (alone/social), study (real-life/VR) and their interaction treatment × study as the predictors. The results revealed no significant effects, treatment (alone/social) (*B* = 0.34, *p* = 0.19), study (real-life/VR) (*B* = −0.10, *p* = 0.73), treatment × study (*B* = −0.032, *p* = 0.94), *R*^*2*^_*m*_ = 0.053.

**Fig. 3:**
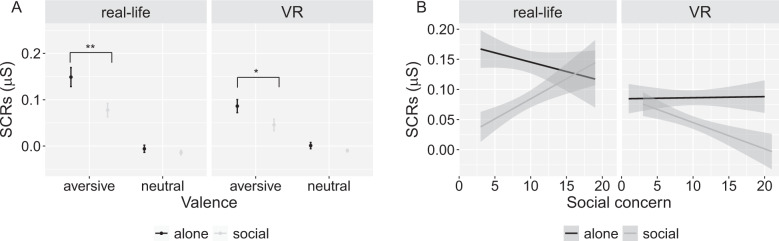
Average skin conductance responses (SCRs) and effect of social concern in the real-life study compared to the virtual-reality (VR) study. **A** Average SCRs to aversive and neutral sounds for the female sample in the alone and the social treatment groups, showing significant social buffering effects in the presence of a real person and a virtual agent. **B** Interaction between treatment (alone/social), study (real-life/VR) and individual social concern scores, reflecting a stronger effect of social concern on the SCRs to aversive sounds in the real-life study compared to the VR study. The values of SCRs were transformed by log (x + 1) to correct the skewness. Error bars indicate standard error. The shade area in (**B**) indicates 95% confidence interval. **p < 0.01, *p < 0.05.

Second, we tested the same model with SCRs to aversive sounds as the dependent variable. The results showed significant main effects of study (*B* = −0.058, *p* = 0.021) and treatment (*B* = −0.065, *p* = 0.003), but no significant study × treatment interaction (*B* = 0.025, *p* = 0.44) (Fig. 3A; Table 5A); *R*^*2*^_*m*_ = 0.030, indicating that the buffering effect was comparable between the real-life and the VR study. Conducting an LMM separately for the VR study revealed a significant main effect of treatment (*B* = −0.040, *p* = 0.033) (Table 5B); *R*^*2*^_*m*_ = 0.035. Post-hoc analysis showed larger SCRs in the alone treatment (*M* = 0.086, SE = 0.013) compared to the social treatment group (*M* = 0.046, SE = 0.013), EMM = 0.040, SE = 0.019. These results indicate a significant social buffering effect in the VR study, which was comparable to the results of female participants in the real-life study.

**Table 5:** Results of the LMM testing the social buffering effects in the real-life and the VR studies.

	*B*	SE	*χ*^*2*^	Df	*P value*
*A LMM testing the two-way interaction effect*					
(Intercept)	0.14	0.016	76.89	1	**<0.001**
Study	−0.058	0.025	5.35	1	**0.021**
Treatment	−0.065	0.022	9.047	1	**0.0026**
PANAS-negative	−0.0057	0.011	0.26	1	0.61
STAI-state-pre	0.012	0.010	1.35	1	0.25
ADS	−0.0049	0.0097	0.25	1	0.62
Study × Treatment	0.025	0.033	0.59	1	0.44
*B LMM testing the buffering effect in VR study*					
(Intercept)	0.086	0.013	41.85	1	**<0.001**
Treatment	−0.040	0.019	4.54	1	**0.033**
PANAS-negative	−0.0034	0.012	0.079	1	0.78
STAI-state-pre	0.023	0.011	4.03	1	**0.045**
ADS	−0.015	0.011	1.83	1	0.18

Bold values are significant at *p* < 0.05.

*PANAS* Positive and Negative Affect Schedule, *STAI-state-pre* state anxiety scores before the experiment based on the State scale of the State-Trait Anxiety Inventory (STAI), *ADS* Allgemeine Depressions Skala, *SE* standard error.

Given the differences in impression regarding the real partner and the virtual agent (Table 4), we tested whether impression ratings significantly predicted individual differences in SCRs in the social treatment groups. An LMM with SCRs to aversive sounds as the dependent variable and study (real-life/VR), impression rating, and study × impression rating as the predictors showed no significant main effects of study (*B* = 0.0058, *p* = 0.82) and impression rating (*B* = 0.013, *p* = 0.39), and no significant study × impression rating interaction (*B* = 0.024, *p* = 0.32); *R*^*2*^_*m*_ = 0.029, indicating that differences in impression ratings did not predict the SCRs in the social treatment groups.

#### Virtual social buffering occurs independently of social concern

Next, we tested if the social buffering of fear in the presence of a virtual person is shaped by social concern. The LMM with social concern, treatment (social/alone), study (real-life/VR) and their interaction items as the predictors, SCRs to aversive sounds as the dependent variable showed significant main effects of treatment (*B* = −0.061, *p* = 0.005) and study (*B* = −0.059, *p* = 0.018), a significant two-way interaction between social concern and treatment (*B* = 0.049, *p* = 0.035), and a significant three-way interaction between social concern, treatment and study (*B* = −0.070, *p* = 0.032), *R*^*2*^_*m*_ = 0.039 (Fig. 3B; Table 6). This indicates that the reduction in SCRs in the social treatment group decreased with social concern in the real-life study, while there was no such effect in the VR study (*B* = −0.029, *p* = 0.086; Fig. 3B). According to these results, social concern did not modulate the buffering effect in the VR study.

**Table 6:** Results of the LMM testing the effect of social concern on buffering in the presence of a real-life and a virtual person.

	*B*	SE	*χ*^*2*^	Df	*P value*
(Intercept)	0.14	0.01634	76.45	1	**<0.001**
Study	−0.059	0.025	5.62	1	**0.018**
Treatment	−0.061	0.022	7.99	1	**0.0047**
Social concern	−0.018	0.017	1.14	1	0.29
PANAS-negative	−0.0037	0.012	0.10	1	0.75
STAI-state-pre	0.016	0.011	2.28	1	0.13
ADS	−0.0062	0.0097	0.40	1	0.53
Study × Treatment	0.024	0.032	0.53	1	0.47
Study × Social concern	0.015	0.022	0.49	1	0.49
Treatment × Social concern	0.049	0.023	4.45	1	**0.035**
Study × Treatment × Social concern	−0.070	0.033	4.61	1	**0.032**

Bold values are significant at *p* < 0.05.

*PANAS* Positive and Negative Affect Schedule, *STAI-state-pre* state anxiety scores before the experiment based on the State scale of the State-Trait Anxiety Inventory (STAI), *ADS* Allgemeine Depressions Skala, *SE* standard error.

In addition, we conducted the same analysis on the other ASI-3 subscales: physical concern and cognitive concern. Both LMMs on physical concern and on cognitive concern showed no significant effects (all *p* > 0.05, Tables S11 and S12), indicating no modulating effect of cognitive concern and physical concern.

## Discussion

The results of our first study revealed an increased social buffering effect in females (Fig. 2A) that is mainly driven by those participants that scored low on social concern (Fig. 2B). In males, social concern increased the SCRs to aversive sounds in the alone treatment group (Fig. 2B). However, a similar increase in SCRs in the male sample was also observed with increasing physical concern (Fig. S1), indicating that in the male sample, social concern had no specific effect. Importantly, the differential effects of social concern on social buffering in females and males were found although both samples had a comparable average social concern score (Table 1). This means that females scored as high (or low) on social concern as males, but, in contrast to males, individual social concern affected the individual extent of female-social buffering.

Previous studies have shown that increases in trait and state anxiety are related to increasing physiological responses, and a stronger attenuation of these responses in the presence of others, i.e., stronger social buffering effects [9, 12, 13]. Trait anxiety assessed by the STAI [40] covers the individual disposition to respond in an anxious way, and STAI-state anxiety reflects a transitory emotion characterized by physiological arousal and consciously perceived feeling of apprehension, dread, and tension [40]. Social concern as measured by the social concern scale of the ASI [22] reflects the fear of publicly observable anxious reactions, and thus targets a more specific anxiety dimension than the STAI. To account for the potential effects of state and trait anxiety, the individual STAI state and trait scores were added as control variables in analyses testing social concern effect. According to our results, social concern scores predicted a reduction of the social buffering effect in real-life study, even if individual differences in state and trait anxiety were taken into account (Tables S13, S14), highlighting the relevance of social concern for explaining individual differences in social buffering in females.

The differential effect of social concern in females and males supports previous evidence, suggesting that females are affected by social anxiety in a large variety of social situations [29, 30], while males are specifically affected in mating contexts, i.e., typically in the presence of a female [31]. Building upon these previous results, our findings showed that individual differences in social concern can account for gender differences in social buffering.

That said, our study showed significant social buffering in female participants in the presence of a female partner, raising the question whether the observed social buffering effect was driven by the presence of a female partner. Impression ratings were more positive for the female partner compared to the male partner, but did not account for the observed gender effect in social buffering. Given that the gender-related differences in impression ratings was the only difference between the social treatments with a female and a male partner, it is unlikely that the observed social buffering effects were mainly driven by the gender of the partner. However, future studies should systematically test for the effect of the partner’s sex by conducting the same social treatment conditions with partners from the opposite sex (i.e., to assess social buffering in females in the presence of a male partner and social buffering in males in the presence of a female partner).

When experiencing the aversive sound alone, females showed higher SCRs compared to males (Fig. 2A), consistent with previous findings indicating that females respond more strongly to aversive events than males [55–57]. However, this gender difference in SCRs in the alone treatment groups cannot account for the differential effect of social concern on SCRs in the social treatment groups between females and males in Study 1, and between real and virtual social presence (comparison of Study 1 and Study 2).

In more detail, the results of Study 2 showed that females also benefit from the presence of a virtual person, indicated by a reduction of fear-related SCRs in the presence of a virtual agent (Fig. 3A). In contrast to social buffering in the presence of a real person, the virtual social buffering effect occurred independently of individual social concern scores (Fig. 3B), indicating that a virtual agent can be a safety cue for females with high social concern. The finding of social buffering effects in the VR environment is in line with previous studies showing a reduction of fear responses [28] or social stress [27] if participants believed that the agent was controlled by a real person, such as a romantic partner [28].

Extending these studies, our results showed social buffering of fear in females in the mere presence of a virtual person, i.e., an agent that was facing away and that was not controlled by a human agent. According to their ratings on the NMMSP questionnaire [53], our participants perceived the virtual agent as present, but not as a social interaction partner. Despite this rather minimalistic virtual social treatment, we observed social buffering in females irrespective of individual differences in social concern. This finding is interesting in two ways. First, it bolsters the claim that at least in females the mere presence of another individual can buffer autonomic fear responses [9]. Second, our findings indicate that females scoring high on social concern can benefit from virtual social presence, although they show reduced social buffering in the presence of a real person.

In our study, we investigated social buffering of human fear in females and males in the presence of a real person and in the presence of a virtual representation of this person, using an identical paradigm. Based on this systematic approach, we could directly compare how social concern affects the social buffering of human fear across genders, and in different environments (real-life vs VR). On the theoretical level, our findings provide novel insights into individual differences that shape the social modulation of autonomic human fear responses. Our finding that the mere virtual presence of another person can attenuate autonomic fear responses in females independently of individual differences in social concern is of potential practical relevance for the treatment of anxiety disorders. In future studies, it would be interesting to contrast these effects with the social presence on the processing of positive stimuli.

## Supplementary information

Supplemental material

Supplemental figure

## Data Availability

All Data and scripts of both studies are provided at https://osf.io/zcu67/?view_only=0d4449de234d45e99adfea1c18f22b26.
