# Supplementary material for: Social buffering of human fear is shaped by gender, social concern, and the presence of real vs virtual agents
Source: Transl Psychiatry. 2021 Dec 20;11:641. doi: 10.1038/s41398-021-01761-5 (PMC8688413; doi:10.1038/s41398-021-01761-5)
Supplement: Supplementary file 1 — Supplemental material [file 41398_2021_1761_MOESM1_ESM.docx]

**Supplementary Results**

**Table S1**. Results of LMM with SCRs to aversive cue as the dependent variable testing the social buffering effect across genders.

|  | ***B*** | ***SE*** | ***χ^2^*** | ***Df*** | ***p*** |
| --- | --- | --- | --- | --- | --- |
| (Intercept) | -.012 | .0088 | 1.80 | 1 | .18 |
| Treatment | -.017 | .012 | 2.17 | 1 | .14 |
| Gender | -.0022 | .012 | .034 | 1 | .85 |
| PANAS-negative | .0008 | .0054 | .021 | 1 | .88 |
| STAI-state-pre | .0030 | .0057 | .28 | 1 | .60 |
| ADS | -.0066 | .0052 | 1.61 | 1 | .20 |
| Treatment × Gender | .028 | .017 | 2.78 | 1 | .096 |

***Note.*** PANAS: [Positive and Negative Affect Schedule;](https://bmcpsychiatry.biomedcentral.com/articles/10.1186/s12888-020-2472-1) STAI-state-pre: state anxiety scores before the experiment based on the State scale of the State-Trait Anxiety Inventory (STAI); ADS: Allgemeine Depressions Skala; SE: standard error. Bold values are significant at *p* < .05.

**Table S2.** Results of the LMM with SCRs to aversive cue as the dependent variable testing the social buffering effect across studies.

|  | ***B*** | ***SE*** | ***χ^2^*** | ***Df*** | ***p*** |
| --- | --- | --- | --- | --- | --- |
| (Intercept) | -.014 | .0095 | 2.28 | 1 | .13 |
| Treatment | -.017 | .013 | 1.84 | 1 | .17 |
| Study | .0016 | .015 | .012 | 1 | .91 |
| PANAS-negative | -.0049 | .0065 | .57 | 1 | .45 |
| STAI-state-pre | .0037 | .0061 | .38 | 1 | .54 |
| ADS | -.0048 | .0056 | .73 | 1 | .39 |
| Treatment × Study | .014 | .019 | .52 | 1 | .47 |

***Note.*** PANAS: [Positive and Negative Affect Schedule;](https://bmcpsychiatry.biomedcentral.com/articles/10.1186/s12888-020-2472-1) STAI-state-pre: state anxiety scores before the experiment based on the State scale of the State-Trait Anxiety Inventory (STAI); ADS: Allgemeine Depressions Skala; SE: standard error. Bold values are significant at *p* < .05.

**Table S3.** Results of the LMM with SCRs to aversive sounds as the dependent variable testing the social buffering effect across genders with SCRs elicited by cue as a covariate.

|  | ***B*** | ***SE*** | ***χ^2^*** | ***Df*** | ***P*** |
| --- | --- | --- | --- | --- | --- |
| (Intercept) | .14 | .015 | 85.76 | 1 | **< .001** |
| Treatment | -.063 | .021 | 9.39 | 1 | **.0022** |
| Gender | -.058 | .021 | 7.47 | 1 | **.0063** |
| SCRs during cue presentation | .0047 | .0031 | 2.36 | 1 | .12 |
| PANAS-negative | -.0049 | .0094 | .28 | 1 | .60 |
| STAI-state-pre | .0018 | .0098 | .034 | 1 | .85 |
| ADS | .0001 | .0091 | .0002 | 1 | .99 |
| Treatment × Gender | .057 | .029 | 3.93 | 1 | **.048** |

***Note.*** PANAS: [Positive and Negative Affect Schedule;](https://bmcpsychiatry.biomedcentral.com/articles/10.1186/s12888-020-2472-1) STAI-state-pre: state anxiety scores before the experiment based on the State scale of the State-Trait Anxiety Inventory (STAI); ADS: Allgemeine Depressions Skala; SE: standard error. Bold values are significant at *p* < .05.

**Table S4.** Results of the LMM with SCRs to aversive sounds as the dependent variable testing the effect of social concern on social buffering effect across genders with SCRs elicited by cue as a covariate.

|  | ***B*** | ***SE*** | ***χ^2^*** | ***Df*** | ***P*** |
| --- | --- | --- | --- | --- | --- |
| (Intercept) | .14 | .015 | 88.45 | 1 | **< .001** |
| Treatment | -.062 | .020 | 9.78 | 1 | **.0018** |
| Gender | -.055 | .020 | 7.24 | 1 | **.0071** |
| Social concern | -.018 | .014 | 1.56 | 1 | .21 |
| SCRs during cue presentation | .0047 | .0031 | 2.33 | 1 | .13 |
| PANAS-negative | -.0094 | .0092 | 1.04 | 1 | .31 |
| STAI-state-pre | .0028 | .0097 | .084 | 1 | .77 |
| ADS | .0035 | .0091 | .15 | 1 | .70 |
| Treatment × Gender | .057 | .028 | 4.19 | 1 | **.041** |
| Treatment × Social concern | .045 | .020 | 5.28 | 1 | **.022** |
| Gender × Social concern | .053 | .020 | 6.78 | 1 | **.0092** |
| Treatment × Gender × Social concern | -.079 | .028 | 7.79 | 1 | **.0053** |

***Note.*** PANAS: [Positive and Negative Affect Schedule;](https://bmcpsychiatry.biomedcentral.com/articles/10.1186/s12888-020-2472-1) STAI-state-pre: state anxiety scores before the experiment based on the State scale of the State-Trait Anxiety Inventory (STAI); ADS: Allgemeine Depressions Skala; SE: standard error. Bold values are significant at *p* < .05.

**Table S5.** Results of the LMM with SCRs to aversive sounds as the dependent variable testing the social buffering effect across studies with SCRs elicited by cue as a covariate.

|  | ***B*** | ***SE*** | ***χ^2^*** | ***Df*** | ***p*** |
| --- | --- | --- | --- | --- | --- |
| **a. LMM testing the two-way interaction effect** | | | | | |
| (Intercept) | .14 | .016 | 76.43 | 1 | **< .001** |
| Treatment | -.064 | .022 | 8.75 | 1 | **.0031** |
| Study | -.057 | .025 | 5.27 | 1 | **.022** |
| SCRs during cue presentation | .0007 | .0036 | .037 | 1 | .85 |
| PANAS-negative | -.0065 | .011 | .34 | 1 | .56 |
| STAI-state-pre | .013 | .010 | 1.65 | 1 | .20 |
| ADS | -.0044 | .0097 | .20 | 1 | .65 |
| Treatment × Study | .025 | .033 | .56 | 1 | .45 |
| **b. LMM testing the buffering effect in VR study** | | | | | |
| (Intercept) | .086 | .013 | 41.72 | 1 | **< .001** |
| Treatment | -.040 | .019 | 4.46 | 1 | **.035** |
| SCRs during cue presentation | -.0076 | .0039 | 3.77 | 1 | .052 |
| PANAS-negative | -.0033 | .012 | .08 | 1 | .78 |
| STAI-state-pre | .023 | .011 | 4.00 | 1 | **.045** |
| ADS | -.015 | .011 | 1.88 | 1 | .17 |

***Note.*** PANAS: [Positive and Negative Affect Schedule;](https://bmcpsychiatry.biomedcentral.com/articles/10.1186/s12888-020-2472-1) STAI-state-pre: state anxiety scores before the experiment based on the State scale of the State-Trait Anxiety Inventory (STAI); ADS: Allgemeine Depressions Skala; SE: standard error. Bold values are significant at *p* < .05.

**Table S6.** Results of the LMM with SCRs to aversive sounds as the dependent variable testing the effect of social concern on social buffering effect across studies with SCRs elicited by cue as a covariate.

|  | ***B*** | ***SE*** | ***χ^2^*** | ***Df*** | ***p*** |
| --- | --- | --- | --- | --- | --- |
| (Intercept) | .14 | .016 | 75.86 | 1 | **< .001** |
| Treatment | -.060 | .022 | 7.66 | 1 | **.0057** |
| Study | -.059 | .025 | 5.48 | 1 | **.019** |
| Social concern | -.020 | .017 | 1.43 | 1 | .23 |
| SCRs during cue presentation | .0005 | .0036 | .022 | 1 | .88 |
| PANAS-negative | -.0047 | .012 | .16 | 1 | .69 |
| STAI-state-pre | .017 | .011 | 2.68 | 1 | .10 |
| ADS | -.0056 | .0097 | .33 | 1 | .57 |
| Treatment × Study | .023 | .032 | .49 | 1 | .48 |
| Treatment × Social concern | .051 | .023 | 4.90 | 1 | **.027** |
| Study × Social concern | .017 | .022 | .62 | 1 | .43 |
| Treatment × Study × Social concern | -.072 | .033 | 4.87 | 1 | **.03** |

***Note.*** PANAS: [Positive and Negative Affect Schedule;](https://bmcpsychiatry.biomedcentral.com/articles/10.1186/s12888-020-2472-1) STAI-state-pre: state anxiety scores before the experiment based on the State scale of the State-Trait Anxiety Inventory (STAI); ADS: Allgemeine Depressions Skala; SE: standard error. Bold values are significant at *p* < .05.

**Table S7.** Results of the LMM with SCRs to aversive sounds as the dependent variable testing the effect of habituation on social buffering effect across genders.

|  | ***B*** | ***SE*** | ***χ^2^*** | ***Df*** | ***P*** |
| --- | --- | --- | --- | --- | --- |
| (Intercept) | .20 | .017 | 14.09 | 1 | **< .001** |
| Treatment | -.083 | .022 | 13.82 | 1 | **< .001** |
| Gender | -.077 | .023 | 11.34 | 1 | **< .001** |
| Half | -.10 | .012 | 73.96 | 1 | **< .001** |
| PANAS-negative | -.0039 | .0094 | .18 | 1 | .67 |
| STAI-state-pre | .0006 | .0099 | .0039 | 1 | .95 |
| ADS | -.0005 | .0091 | .0028 | 1 | .96 |
| Treatment × Gender | .060 | .031 | 3.66 | 1 | .056 |
| Treatment × Half | .035 | .016 | 4.64 | 1 | **.031** |
| Gender × Half | .036 | .016 | 4.78 | 1 | **.029** |
| Treatment × Gender × Half | -.0018 | .023 | .0062 | 1 | .94 |

***Note.*** PANAS: [Positive and Negative Affect Schedule;](https://bmcpsychiatry.biomedcentral.com/articles/10.1186/s12888-020-2472-1) STAI-state-pre: state anxiety scores before the experiment based on the State scale of the State-Trait Anxiety Inventory (STAI); ADS: Allgemeine Depressions Skala; SE: standard error. Bold values are significant at *p* < .05.

**Table S8.** Results of the LMM with SCRs to aversive sounds as the dependent variable testing the effect of habituation on social buffering effect across studies.

|  | ***B*** | ***SE*** | ***χ^2^*** | ***Df*** | ***p*** |
| --- | --- | --- | --- | --- | --- |
| (Intercept) | .20 | .018 | 123.56 | 1 | **< .001** |
| Treatment | -.083 | .024 | 12.52 | 1 | **< .001** |
| Study | -.092 | .027 | 11.58 | 1 | **< .001** |
| Half | -.10 | .013 | 61.70 | 1 | **< .001** |
| PANAS-negative | -.0058 | .011 | .27 | 1 | .60 |
| STAI-state-pre | .012 | .010 | 1.37 | 1 | .24 |
| ADS | -.0049 | .0097 | .25 | 1 | .62 |
| Treatment × Study | .052 | .035 | 2.16 | 1 | .14 |
| Treatment × Half | .035 | .018 | 3.87 | 1 | **.049** |
| Study × Half | .066 | .019 | 11.39 | 1 | **< .001** |
| Treatment × Study × Half | -.052 | .027 | 3.79 | 1 | .052 |

***Note.*** PANAS: [Positive and Negative Affect Schedule;](https://bmcpsychiatry.biomedcentral.com/articles/10.1186/s12888-020-2472-1) STAI-state-pre: state anxiety scores before the experiment based on the State scale of the State-Trait Anxiety Inventory (STAI); ADS: Allgemeine Depressions Skala; SE: standard error. Bold values are significant at *p* < .05.

**Table S9.** Results of the LMM with SCRs to aversive sounds as the dependent variable testing the effect of physical concern on social buffering effect across genders.

|  | ***B*** | ***SE*** | ***χ^2^*** | ***Df*** | ***p*** |
| --- | --- | --- | --- | --- | --- |
| (Intercept) | .14 | .015 | 85.94 | 1 | **< .001** |
| Treatment | -.063 | .020 | 9.56 | 1 | **.002** |
| Gender | -.060 | .021 | 8.05 | 1 | **.005** |
| Physical concern | .0099 | .015 | .43 | 1 | .51 |
| PANAS-negative | -.0053 | .0096 | .30 | 1 | .58 |
| STAI-state-pre | -.0012 | .0099 | .0153 | 1 | .90 |
| ADS | -.0014 | .0093 | .0233 | 1 | .88 |
| Treatment × Gender | .062 | .029 | 4.68 | 1 | **.031** |
| Treatment × Physical concern | -.013 | .021 | .43 | 1 | .51 |
| Gender × Physical concern | .023 | .020 | 1.21 | 1 | .27 |
| Treatment × Gender × Physical concern | -.0075 | .029 | .0651 | 1 | .80 |

***Note.*** PANAS: [Positive and Negative Affect Schedule;](https://bmcpsychiatry.biomedcentral.com/articles/10.1186/s12888-020-2472-1) STAI-state-pre: state anxiety scores before the experiment based on the State scale of the State-Trait Anxiety Inventory (STAI); ADS: Allgemeine Depressions Skala; SE: standard error. Bold values are significant at *p* < .05.

**Table S10.** Results of the LMM with SCRs to aversive sounds as the dependent variable testing the effect of cognitive concern on social buffering effect across genders.

|  | ***B*** | ***SE*** | ***χ^2^*** | ***Df*** | ***p*** |
| --- | --- | --- | --- | --- | --- |
| (Intercept) | .14 | .015 | 84.75 | 1 | **< .001** |
| Treatment | -.064 | .021 | 9.58 | 1 | **< .001** |
| Gender | -.058 | .021 | 7.39 | 1 | **< .001** |
| Cognitive concern | .015 | .016 | .92 | 1 | .34 |
| PANAS-negative | -.0045 | .0095 | .22 | 1 | .64 |
| STAI-state-pre | -.0007 | .010 | .0043 | 1 | .95 |
| ADS | -.0012 | .0096 | .015 | 1 | .90 |
| Treatment × Gender | .058 | .029 | 4.02 | 1 | **.045** |
| Treatment × Cognitive concern | -.024 | .020 | 1.42 | 1 | .23 |
| Gender × Cognitive concern | -.0046 | .024 | .036 | 1 | .85 |
| Treatment × Gender × Cognitive concern | .021 | .031 | .4605 | 1 | .50 |

***Note.*** PANAS: [Positive and Negative Affect Schedule;](https://bmcpsychiatry.biomedcentral.com/articles/10.1186/s12888-020-2472-1) STAI-state-pre: state anxiety scores before the experiment based on the State scale of the State-Trait Anxiety Inventory (STAI); ADS: Allgemeine Depressions Skala; SE: standard error. Bold values are significant at *p* < .05.

**Table S11.** Results of the LMM with SCRs to aversive sounds as the dependent variable testing the effect of physical concern on social buffering effect across studies.

|  | ***B*** | ***SE*** | ***χ^2^*** | ***Df*** | ***p*** |
| --- | --- | --- | --- | --- | --- |
| (Intercept) | .14 | .017 | 72.35 | 1 | **< .001** |
| Study | -.057 | .026 | 4.66 | 1 | **.031** |
| Treatment | -.065 | .022 | 8.72 | 1 | **.003** |
| Physical concern | .0046 | .018 | .066 | 1 | .80 |
| PANAS-negative | -.0071 | .012 | .33 | 1 | .57 |
| STAI-state-pre | .0115 | .011 | 1.12 | 1 | .29 |
| ADS | -.0042 | .010 | .17 | 1 | .68 |
| Study × Treatment | .025 | .033 | .54 | 1 | .46 |
| Study × Physical concern | -.0059 | .024 | .060 | 1 | .81 |
| Treatment × Physical concern | -.0096 | .024 | .16 | 1 | .69 |
| Study × Treatment × Physical concern | .015 | .034 | .19 | 1 | .66 |

***Note.*** PANAS: [Positive and Negative Affect Schedule;](https://bmcpsychiatry.biomedcentral.com/articles/10.1186/s12888-020-2472-1) STAI-state-pre: state anxiety scores before the experiment based on the State scale of the State-Trait Anxiety Inventory (STAI); ADS: Allgemeine Depressions Skala; SE: standard error. Bold values are significant at *p* < .05.

**Table S12.** Results of the LMM with SCRs to aversive sounds as the dependent variable testing the effect of cognitive concern on social buffering effect across studies.

|  | ***B*** | ***SE*** | ***χ^2^*** | ***Df*** | ***p*** |
| --- | --- | --- | --- | --- | --- |
| (Intercept) | .15 | .017 | 75.40 | 1 | **< .001** |
| Study | -.061 | .026 | 5.58 | 1 | **.018** |
| Treatment | -.068 | .022 | 9.48 | 1 | **.002** |
| Cognitive concern | .015 | .019 | .64 | 1 | .43 |
| PANAS-negative | -.0053 | .012 | .20 | 1 | .65 |
| STAI-state-pre | .012 | .011 | 1.28 | 1 | .26 |
| ADS | -.0044 | .010 | .19 | 1 | .67 |
| Study × Treatment | .028 | .034 | .72 | 1 | .40 |
| Study × Cognitive concern | -.026 | .027 | .94 | 1 | .33 |
| Treatment × Cognitive concern | -.026 | .024 | 1.22 | 1 | .27 |
| Study × Treatment × Cognitive concern | .037 | .034 | 1.15 | 1 | .28 |

***Note.*** PANAS: [Positive and Negative Affect Schedule;](https://bmcpsychiatry.biomedcentral.com/articles/10.1186/s12888-020-2472-1) STAI-state-pre: state anxiety scores before the experiment based on the State scale of the State-Trait Anxiety Inventory (STAI); ADS: Allgemeine Depressions Skala; SE: standard error. Bold values are significant at *p* < .05.

**Table S13.** Results of the LMM with SCRs to aversive sounds as the dependent variable testing the effect of social concern on social buffering effect across genders, controlling for individual differences in state and trait anxiety.

|  | ***B*** | ***SE*** | ***χ^2^*** | ***Df*** | ***p*** |
| --- | --- | --- | --- | --- | --- |
| (Intercept) | .14 | .015 | 87.46 | 1 | **<.001** |
| Treatment | -.064 | .020 | 1.19 | 1 | **.001** |
| Gender | -.055 | .021 | 7.09 | 1 | **.008** |
| Social concern | -.016 | .014 | 1.23 | 1 | .27 |
| PANAS-negative | -.0095 | .0094 | 1.02 | 1 | .31 |
| ADS | .0041 | .010 | .17 | 1 | .68 |
| STAI-trait anxiety | -.0045 | .011 | .18 | 1 | .68 |
| STAI-state-pre | .0014 | .011 | .016 | 1 | .90 |
| STAI-state-post | .0071 | .0080 | .79 | 1 | .37 |
| Treatment × Gender | .059 | .028 | 4.37 | 1 | **.036** |
| Treatment × Social concern | .043 | .020 | 4.56 | 1 | **.033** |
| Gender × Social concern | .054 | .021 | 6.71 | 1 | **.010** |
| Treatment × Gender × Social concern | -.077 | .029 | 7.25 | 1 | **.007** |

***Note.*** PANAS: [Positive and Negative Affect Schedule;](https://bmcpsychiatry.biomedcentral.com/articles/10.1186/s12888-020-2472-1) ADS: Allgemeine Depressions Skala; STAI-trait: trait anxiety scores based on the trait scale of the State-Trait Anxiety Inventory (STAI); STAI-state-pre: state anxiety scores before the experiment based on the State scale of the State-Trait Anxiety Inventory (STAI); STAI-state-post: state anxiety scores after the experiment based on the state scale of the State scale of the State-Trait Anxiety Inventory (STAI); SE: standard error. Bold values are significant at *p* < .05.

**Table S14.** Results of the LMM with SCRs to aversive sounds as the dependent variable testing the effect of social concern on social buffering effect across studies, controlling for individual differences in state and trait anxiety.

|  | ***B*** | ***SE*** | ***χ^2^*** | ***Df*** | ***P*** |
| --- | --- | --- | --- | --- | --- |
| (Intercept) | .14 | .017 | 73.69 | 1 | **<.001** |
| Study | -.058 | .026 | 5.08 | 1 | **.024** |
| Treatment | -.061 | .022 | 7.91 | 1 | **.005** |
| Social concern | -.019 | .017 | 1.21 | 1 | .27 |
| PANAS-negative | -.0040 | .012 | .11 | 1 | .74 |
| ADS | -.0078 | .011 | .51 | 1 | .47 |
| STAI-trait anxiety | .0024 | .012 | .041 | 1 | .84 |
| STAI-state-pre | .013 | .012 | 1.25 | 1 | .26 |
| STAI-state-post | .0052 | .0095 | .30 | 1 | .58 |
| Study × Treatment | .024 | .033 | .54 | 1 | .46 |
| Study × Social concern | .015 | .022 | .46 | 1 | .50 |
| Treatment × Social concern | .049 | .023 | 4.31 | 1 | **.038** |
| Study × Treatment × Social concern | -.071 | .033 | 4.57 | 1 | **.033** |

***Note.*** PANAS: [Positive and Negative Affect Schedule;](https://bmcpsychiatry.biomedcentral.com/articles/10.1186/s12888-020-2472-1) ADS: Allgemeine Depressions Skala; STAI-trait: trait anxiety scores based on the trait scale of the State-Trait Anxiety Inventory (STAI); STAI-state-pre: state anxiety scores before the experiment based on the State scale of the State-Trait Anxiety Inventory (STAI); STAI-state-post: state anxiety scores after the experiment based on the state scale of the State scale of the State-Trait Anxiety Inventory (STAI); SE: standard error. Bold values are significant at *p* < .05.

**Figure legend**

Figure S1. The modulating effect of social concern (A) and physical concern (B) on social buffering effect in males. Males showed increasing SCRs (skin conductance responses) with increasing social concern and physical concern in alone treatment group, indicating an unspecific increase in SCRs with increasing anxiety.
