# Supplementary figures and images for: Social buffering of human fear is shaped by gender, social concern, and the presence of real vs virtual agents
Source: Transl Psychiatry. 2021 Dec 20;11:641. doi: 10.1038/s41398-021-01761-5 (PMC8688413; doi:10.1038/s41398-021-01761-5)

**A**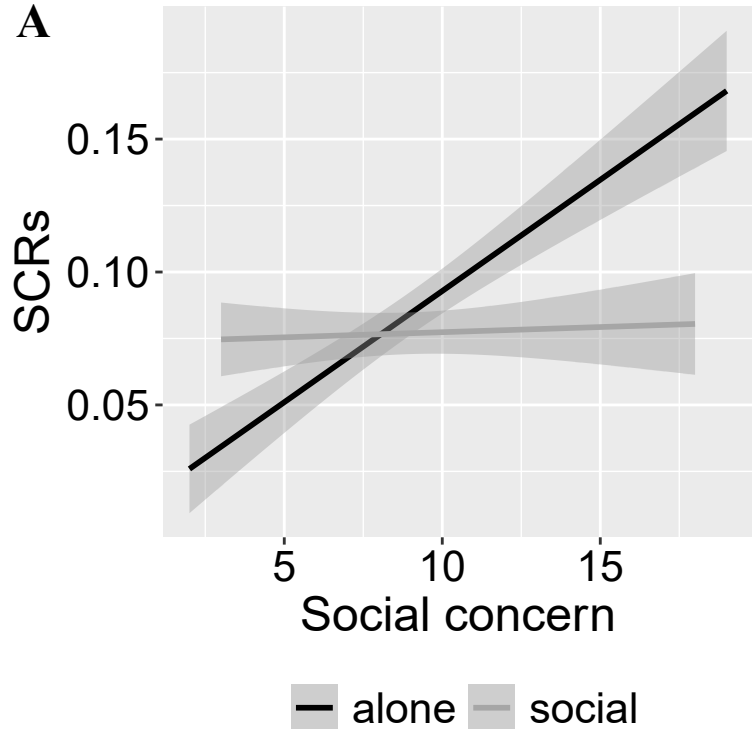**B**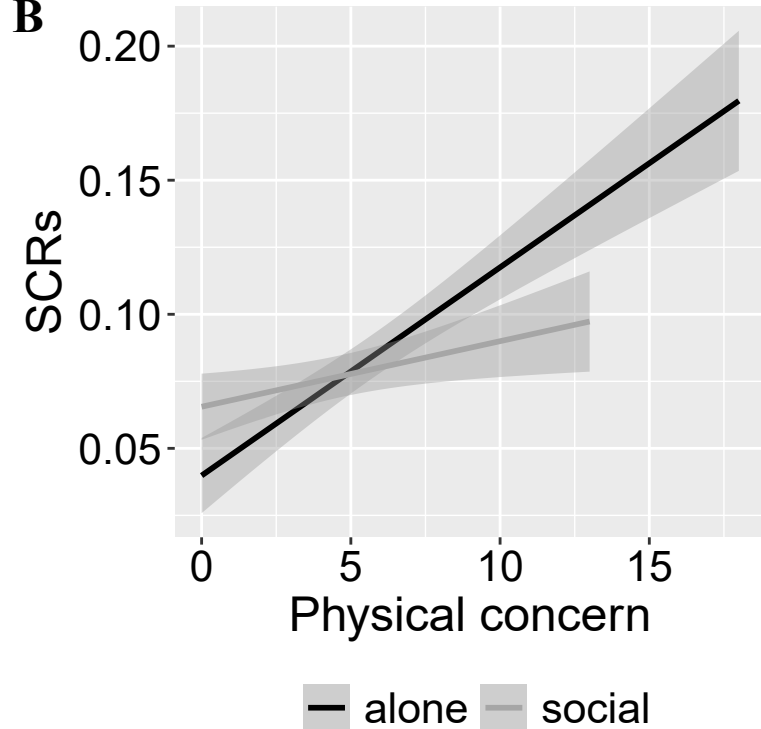

Supplement: Supplementary file 2 — Supplemental figure [file 41398_2021_1761_MOESM2_ESM.pdf]
